# Detection of Specific IgG-Antibodies Against *Toxoplasma gondii* in the Serum and Milk of Domestic Donkeys During Lactation in China: A Potential Public Health Concern

**DOI:** 10.3389/fcimb.2021.760400

**Published:** 2021-10-21

**Authors:** Long Chen, Zi-Jian Zhao, Qing-Feng Meng

**Affiliations:** ^1^ Institute of Animal Nutrition and Feed, Jilin Academy of Agricultural Sciences, Gongzhuling, China; ^2^ Institute of Agro-food Technology, Jilin Academy of Agricultural Sciences, Changchun, China; ^3^ Technology Center, Changchun Customs, Changchun, China

**Keywords:** *Toxoplasma gondii*, specific IgG-antibodies, domestic donkeys, sera, milk

## Abstract

*Toxoplasma gondii* is a worldwide zoonotic protozoan. Donkeys are often susceptible to many pathological agents, acting as carriers of pathogens for other animal species and humans. However, data on the prevalence of *T. gondii* in donkeys during lactation and on the status of antibodies against *T. gondii* in donkey milk are lacking. A cross-sectional study evaluated the variation of the anti-*T. gondii* antibodies in the blood and milk of domestic donkeys during lactation. A total of 418 domestic donkeys were randomly selected from the Shandong province, eastern China from January 2019 to March 2020. The anti-*T. gondii* antibodies were found in 11.72% (49/418) serum and 9.81% (41/418) milk samples using a commercial ELISA kit, respectively. There was a very high consistency between the serum and milk (Spearman’s coefficient = 0.858, *p*-value < 0.0001 and Kendall’s tau = 0.688, *p*-value < 0.0001), particularly at the 45th to 60th day of lactation. The present results of the statistical analysis showed that the history of abortion (*p* = 0.026; adjusted OR = 2.20; 95% CI: 1.15–4.20) and cat in the house (*p* = 0.008; adjusted OR = 2.36; 95% CI: 1.26–4.44) were significantly associated with *T. gondii* infection in the domestic donkeys. This is the first report to detect antibodies against *T. gondii* in donkey milk in China. These results indicate a potential risk of humans contracting the infection through the consumption of raw milk from the naturally infected donkeys.

## Introduction

Toxoplasmosis is a very important and prevalent foodborne parasitic disease, caused by *Toxoplasma gondii*, infecting all warm-blooded animals including human beings, livestock, birds, and marine mammals (Dubey, 2010). Normally, *T. gondii* infection does not result in obvious clinical symptoms. However, the *T. gondii* infection occurring in pregnant women, organ transplant patients, and patients with immune deficiency triggers severe clinical symptoms and even death (Montoya and Liesenfeld, 2004). Thus, *T. gondii* infection induces huge damages in both the public health sector and the veterinary field. The infection occurs mainly in three ways: congenital transmission, organ transplant/blood transfusion, and through food and water contaminated by either of the three forms of this parasite (tachyzoite, cysts, and oocysts) (Tenter et al., 2000). Usually, raw or undercooked meat, contaminated milk, and unwashed fruit vegetables can induce this parasitic infection (Pinto-Ferreira et al., 2019). To date, no reports suggest evidence of *T. gondii* infection due to the consumption of donkey’s milk, and raw goat’s milk has been proven to be associated with the *T. gondii* infection in humans in clinical practice (Camossi et al., 2011).

So far, *T. gondii* has been reported in the milk of various hosts like a goat (Bezerra et al., 2015; Gazzonis et al., 2019), sheep (Iacobucci et al., 2019), cat (Powell et al., 2001), camel (Saad et al., 2018), buffalo (Dehkordi et al., 2013), cow (Koethe et al., 2017), and even lactating women (Azab et al., 1992). Thus, *T. gondii* infection is presumed to occur upon the consumption of either of the milk when consumed raw (Boughattas, 2017). Therefore, there is a necessity of identifying the parasitic contamination in donkey’s milk (Martini et al., 2014). However, there is limited information available on the prevalence of *T. gondii* in donkey’s milk available worldwide (Haridy et al., 2010; Mancianti et al., 2014; Martini et al., 2014; Perrucci et al., 2021), especially in China, which is one of the world’s largest donkey breeding countries.

The consumption of raw milk products has been well-known to pose a very large potential risk, especially in some special groups, such as infants and the aged. Thus, this study aimed to evaluate the prevalence of *T. gondii* in the serum and milk of domestic donkeys during lactation in China. This would provide primary data regarding the prevalence of *T. gondii* in donkey milk in China and add some new data for the safety of the public.

## Materials and Methods

### Ethical Statement

The owners of the donkeys and the local veterinarians were employed to collect the serum and milk from the domestic donkeys. All of the samples were procured with the approval of the owners. All the procedures involving animals were approved by the Animal Care and Ethics Committee of Jilin Academy of Agricultural Sciences.

### Sample and Animal Data Collection

**Figure 1:**
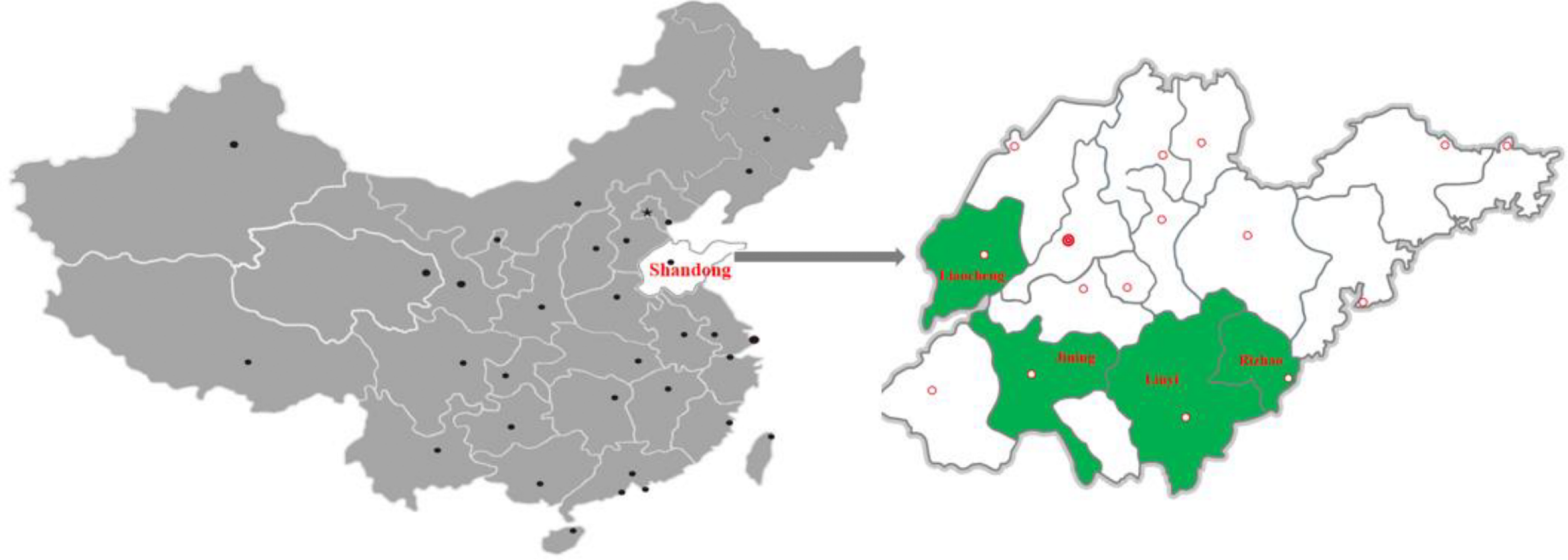
A map of China showing the four cities, Jining, Linyi, Rizhao, and Liaocheng, in Shandong province, eastern China, where the serum and milk samples of the domestic donkeys were collected.

A cross-sectional study was carried out in four donkey culturing cities (Jining, Linyi, Rizhao, and Liaocheng) from the Shandong province, eastern China (**Figure 1**). A total of 418 serum and 418 milk samples from the domestic donkeys were randomly collected from January 2019 to March 2020. The blood samples and corresponding milk samples were obtained from each of the donkeys. About 10 ml of blood samples was obtained from the jugular vein of the donkeys using the blood lancet and stored in vacuum tubes without anticoagulant agents. Before collecting the milk samples, the teats were firstly disinfected, and then, about 10 ml of milk samples was collected by milking donkeys by humans and stored in sterile tubes. After transferring the samples to the laboratory, the blood samples were centrifuged at 1,500 *g* for 10 min and then placed at room temperature for 4 h. Finally, the obtained serum was stored at −20°C until further use. For processing the collected milk samples, the fatty components and the somatic cells were removed according to a previous study (Petruzzelli et al., 2013) and then stored at −20°C until further use. For collecting the animal data, the individual data about the age and history of abortion of each donkey, cats in the house, source of water, and source of fodder were obtained from the owners. Moreover, the day of birth of each donkey was set as day 0, and the day of lactation was calculated (Gazzonis et al., 2019).

### Laboratory Testing for the *T. gondii* Antibody

To detect the specific IgG-antibodies against *T. gondii* in the collected samples, the available commercial ELISA kit (ID Screen^®^ Toxoplasmosis Indirect MultiSpecies, IDVET, Montpellier) was employed according to the instructions of the manufacturer following the protocol described in the previous study (Gazzonis et al., 2018). The absorbance was measured as the optical density (OD) at 450 nm using a microplate reader (BIO-RAD iMark, United States). The test results were calculated according to the formula provided by the manufacturer:


S/P%=100×(OD sample−OD negative control)/(OD positive control−OD negative control).

The cutoff value for the positive serum samples and milk samples were set at S/P% ≥50% and S/P% ≥21.8%, respectively (Gazzonis et al., 2018).

### Statistical Analysis

The statistical analysis was performed using the SPSS 25.0 software package IBM, (Armonk, NY, United States). *p*-values less than 0.05 were considered statistically significant. Spearman and Kendall’s rank correlation coefficients analyzed the correspondence between sera and milk results. The logistic regression was used to analyze the association between the *T. gondii* infection and potential risk factors. The multivariate logistic analysis was further performed using the full model, including all the potential risk factors in the analyses.

## Results

### The *T. gondii* Antibody Detection in the Serum and Milk Samples

In total, 11.72% (49/418) serum samples and 9.81% (41/418) milk samples were found to be positive for the anti-*T. gondii* antibodies, respectively. Comparing the results obtained from the serum and the milk samples, eight positive serum samples were found to have yielded negative results for the correspondent milk samples, while none of the negative serum samples yielded positive correspondent milk samples.

**Figure 2:**
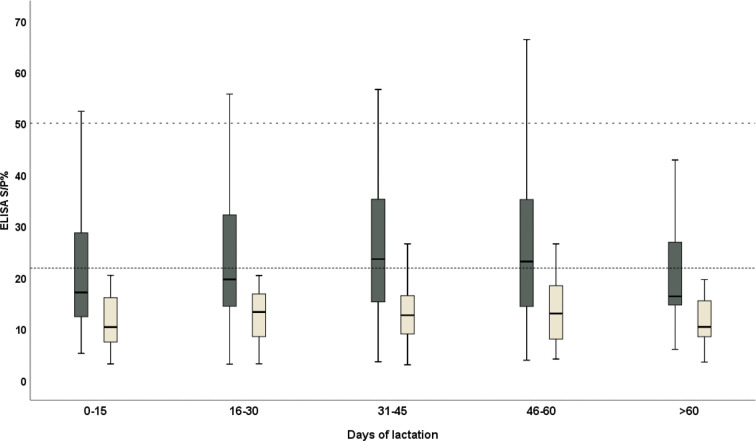
The distribution trend of the ELISA S/P% values of serum (black) and milk (yellow) samples of the domestic donkeys during the lactation. The cutoff values for the anti-*T. gondii* IgG were 50 (dashed line) and 21.8 (dotted line) in the serum and milk samples and were considered positive, respectively.

There was a very high consistency between the results on the serum and milk samples (Spearman’s coefficient = 0.858, *p*-value < 0.0001 and Kendall’s tau = 0.688, *p*-value < 0.0001). The best agreement was obtained from the 46–60 DP (days from parturition), followed by 0–15 DP, while the worst was evident at the second half of the month of lactation (16–30 DP) (**Table 1**). The trend in the antibody level in the serum and milk was explored: the ELISA S/P% values of the serum and milk samples were high in the third phase of lactation (31–45 DP) and the fourth phase of lactation (46–60 DP), respectively. Moreover, both the ELISA S/P% values of the serum and milk samples decreased in the last lactation (>60 DP) (**Figure 2**).

**Table 1:** The conformance between the lactating donkey’s serum and milk samples based on the ELISA S/P% results.

Statistical test	Days from parturition				
	0–15	16–30	31–45	45–60	>60
Kendall’s Tau	0.670	0.665	0.683	0.730	0.649
(*p*-value)	(0.000)	(0.000)	(0.000)	(0.000)	(0.000)
Spearman’s coefficient	0.852	0.833	0.842	0.888	0.836
(*p*-value)	(0.000)	(0.000)	(0.000)	(0.000)	(0.000)

All of the tested domestic donkeys were divided into four age groups. The highest seroprevalence of *T. gondii* in the serum samples was 13.73% for the age group 37–48 months old, and the highest prevalence of *T. gondii* in the milk samples was 11.94% for the age group >48 months old (**Table 2**). In terms of region, Linyi (15.31%) and Rizhao (11.34%) were found to have the highest prevalence of *T. gondii* in the serum and milk samples, respectively (**Table 2**). Considering the sampling time, both the highest prevalence of *T. gondii* in the serum and milk samples were found in winter (16.16% and 14.14, respectively), and the lowest was found in autumn (8.33% and 6.82%. respectively) (**Table 2**). By days from postpartum, the highest prevalence of *T. gondii* in the serum and milk samples were found in the 46–60 DP group (18.67%) and the >60 DP group (15.00%), respectively, but both the lowest prevalence of *T. gondii* in the serum and milk samples were found in the 0–15 DP group (4.29% and 2.86%. respectively) (**Table 2**).

**Table 2:** Univariate analysis of the variables associated with *T. gondii* prevalence in the serum and milk samples of the domestic donkeys tested by ELISA.

Variable	No. tested	Serum			Milk		
		Positivity (%)	Odds ratio (95% confidence interval)	*p*-value	Positivity (%)	Odds ratio (95% confidence interval)	*p*-value
Age (Months)							
≦ 24	67	4.48	0.35 (0.09–1.37)	0.130	4.48	0.35 (0.09–1.37)	0.130
25–36	182	13.19	1.12 (0.48–2.63)	0.794	9.89	0.81 (0.33–1.96)	0.639
37–48	102	13.73	1.17 (0.46–2.97)	0.736	11.76	0.98 (0.38–2.55)	0.972
>48	67	11.94	Reference		11.94	Reference	
Region							
Jining	116	9.48	0.91 (0.38–2.21)	0.842	7.76	0.82 (0.32–2.09)	0.672
Linyi	98	15.31	1.58 (0.69–3.62)	0.283	11.22	1.23 (0.50–3.03)	0.658
Rizhao	97	12.37	1.23 (0.52–2.94)	0.638	11.34	1.24 (0.50–3.07)	0.640
Liaocheng	107	10.28	Reference		9.35	Reference	
Sampling time							
Spring	108	11.11	0.65 (0.29–1.45)	0.291	7.41	0.49 (0.19–1.21)	0.122
Summer	79	12.66	0.75 (0.32–1.76)	0.512	12.66	0.88 (0.37–2.10)	0.774
Autumn	132	8.33	0.47 (0.21–1.07)	0.071	6.82	0.44 (0.18–1.07)	0.071
Winter	99	16.16	Reference		14.14	Reference	
History of abortion							
Yes	130	17.69	2.17 (1.18–3.96)	0.012*	12.31	1.48 (0.76–2.87)	0.251
No	288	9.03	Reference		8.68	Reference	
Days from postpartum							
0–15	70	4.29	0.25 (0.05–1.37)	0.111	2.86	0.17 (0.03–1.08)	0.060
16–30	164	14.02	0.92 (0.25–3.41)	0.906	11.59	0.74 (0.20–2.77)	0.658
31–45	89	6.74	0.41 (0.09–1.80)	0.238	6.74	0.41 (0.09–1.80)	0.238
46–60	75	18.67	1.30 (0.34–5.51)	0.704	14.67	0.97 (0.24–3.89)	0.970
>60	20	15.00	Reference		15.00	Reference	
Cats in house							
Yes	115	20.00	2.66 (1.45–4.90)	0.002*	14.78	2.02 (1.04–3.91)	0.038*
No	303	8.58	Reference		7.92	Reference	
Source of Water							
Well	141	10.64	0.71 (0.35–1.45)	0.344	9.22	0.78 (0.36–1.69)	0.781
Tap water	138	10.14	0.67 (0.32–1.39)	0.284	8.70	0.73 (0.33–1.61)	0.438
Well/Tap water	139	14.39	Reference		11.51	Reference	
Source of fodder							
Forage	96	13.54	1.17 (0.57–2.39)	0.676	13.54	1.79 (0.83–3.85)	0.138
Commercial feed	111	9.91	0.82 (0.39–1.73)	0.600	9.91	1.26 (0.57–2.78)	0.576
Forage/Commercial feed	211	11.85	Reference		8.06	Reference	
Total	418	11.72			41	9.81	

*Statistically significant.

### Risk Factors for *T. gondii* Infection

In the univariate analysis for the serum samples, two variables were found to be associated with the anti-*T. gondii* IgG positivity, including the history of abortion (*p* = 0.012; adjusted OR = 2.17; 95% CI: 1.18–3.96) and cat in the house (*p* = 0.002; adjusted OR = 2.66; 95% CI: 1.45–4.90). Only one variable (cat in the house, *p* = 0.038; adjusted OR = 2.02; 95% CI: 1.04–3.91) was found to be associated with the anti-*T. gondii* IgG positivity in the univariate analysis for the milk samples (**Table 2**). The following multivariate logistic regression showed that the history of abortion (*p* = 0.026; adjusted OR = 2.20; 95% CI: 1.15–4.20) and cat in the house (*p* = 0.008; adjusted OR = 2.36; 95% CI: 1.26–4.44) were independent risk factors for *T. gondii* seropositivity in the domestic donkeys (**Table 3**).

**Table 3:** Multivariate logistic regression with a full model for the risk factors of *T. gondii* infection in the domestic donkeys in China.

Variable	Odds ratio (95% confidence interval)	*P*-value
Age (months) (≦24 *vs*. >48)	0.45 (0.11–1.88)	0.274
Age (months) (25–36 *vs* >48)	1.60 (0.64–3.99)	0.316
Age (months) (37–48 *vs* >48)	1.27 (0.48–3.35)	0.633
Region (Jining *vs* Liaocheng)	0.83 (0.33–2.11)	0.696
Region (Linyi *vs*. Liaocheng)	2.06 (0.86–4.96)	0.106
Region (Rizhao *vs*. Liaocheng)	1.42 (0.58–3.47)	0.444
Sampling time (Spring *vs*. Winter)	0.69 (0.30–1.57)	0.380
Sampling time (Summer *vs*. Winter)	1.08 (0.43–2.73)	0.866
Sampling time (Autumn *vs*. Winter)	0.38 (0.17–0.89)	0.026
History of abortion	2.20 (1.15–4.20)	0.017
Days from postpartum (0–15 *vs*. >60)	0.17 (0.03–0.96)	0.045
Days from postpartum (16–30 *vs*. >60)	0.79 (0.21–3.01)	0.729
Days from postpartum (31–45 *vs*. >60)	0.43 (0.09–1.91)	0.265
Days from postpartum (46–60 *vs*. >60)	0.99 (0.24–4.03)	0.988
Cat in house	2.36 (1.26–4.44)	0.008
Source of Water (Well *vs*. Well/Tap water)	0.57 (0.27–1.23)	0.152
Source of Water (Tap water *vs*. Well/Tap water)	0.62 (0.29–1.33)	0.217
Source of fodder (Forage *vs*. Forage/Commercial feed)	1.23 (0.58–2.61)	0.594
Source of fodder (Commercial feed *vs*. Forage/Commercial feed)	0.71 (0.33–1.55)	0.391

## Discussion

Donkey’s milk has been used since antiquity mainly for its important medicinal properties as well as nutrient values (Li Q. et al., 2020). It is endowed with the potent ability to regulate the immune system to postpone senility, making it a potentially functional health food for inhibiting the progression of some diseases, such as triple-negative breast tumors (Li Q. et al., 2020), type 2 diabetes (Li Y. et al., 2020), and atherosclerosis (Tafaro et al., 2007). Moreover, donkey’s milk has been recognized as an ideal alternative to human milk because of its total protein and lactose contents, as well as similar fatty acid and protein profiles (Zhang et al., 2021). Owing to these advantages, there has been a booming global demand for the direct consumption of donkey milk. This escalating demand has to be met by simultaneously and chiefly prioritizing the safety of the consumers, especially considering that many consumers often buy donkey milk directly raw from the farms and individual raisers (Boughattas, 2017). The ingestion of unpasteurized milk has been found to have potential risks and sources of *T. gondii* infection for children living in rural areas (Radon et al., 2004). Moreover, consumption of unpasteurized milk also elevates the potential risk factor for toxoplasmosis in females with recurrent pregnancy loss (Rehman et al., 2020). The latest China Statistical Yearbook has reported about 2.53 million donkeys in China in 2018 (Luoyizha et al., 2020). Although several studies have been conducted to detect the prevalence of *T. gondii* infection in the donkeys from the different regions of China (Miao et al., 2013; Yang et al., 2013; Zhang et al., 2017; Cong et al., 2018; Meng et al., 2018), the data regarding the prevalence of *T. gondii* infection in the donkey’s milk in China is scarce. This is the first study to estimate the prevalence of the specific IgG-antibodies against *T. gondii* in the milk of the domestic donkeys during lactation in China, which provided important data for controlling and preventing toxoplasmosis in human beings in China.

The present study investigated the anti-*T. gondii* IgG levels during lactation in the serum and milk samples of the domestic donkeys in China and evaluated the information about the dynamics of specific antibody levels both in the serum and milk. About 9.81% (41/418) of milk samples were found to be contaminated with *T. gondii*. Until now, only four studies have been conducted to explore the contamination status of the milk matrix of donkeys by *T. gondii* globally. In Egypt, the antibodies against *T. gondii* in the milk of a pregnant Egyptian donkey female were detected using an ELISA and reported a contamination rate of 46.3% (Haridy et al., 2010). In Italy, *T. gondii* DNA was detected in three of the six tested milk samples using nest-PCR (Mancianti et al., 2014). In another study conducted in Italy, 4 (22.2%) out of 18 donkeys presented *T. gondii* DNA in milk (Martini et al., 2014). Simultaneously, the milk quality in the positive donkeys showed a significant difference compared to that in the negative donkeys, suggesting that *T. gondii* infection might induce changes in the milk quality. Moreover, the DNA of *T. gondii* was found in the milk of three jennies in all the 19 milk samples collected from central Italy by a nest-PCR (Perrucci et al., 2021). In Europe, raw milk collected from any animal can be sold directly to any people (the producer of milk product, a local milk seller, or final consumers) without any processing except refrigeration between 0 and 4°C (Mancianti et al., 2014). To sum up, donkey’s milk should be considered as a potential pathway of *T. gondii* infection in human beings.

The concordance was explored between the serum and milk collected from the different phases of lactation to find the best agreement in the 45–60 days from parturition, followed by the first phase (0–15 days from parturition). However, the phase of lactation was not found to be a risk factor influencing the antibody level both in the serum and milk samples in the present study. Unfortunately, there is limited information about the physiological immunoglobulin levels in the donkey’s milk during lactation. Based on the present data, in milk, the IgG level demonstrates a little change among the different phases of lactation and the peak was evident in the fourth phase of lactation (46–60 DP). Likewise, in the serum, the IgG level was high in the fourth phase of lactation; subsequently, it decreased sharply in the last phase of lactation (>60 DP). However, the trends of antibody levels in the milk samples are mostly the same as those in the serum; thus, the IgG trend of milk during lactation might reflect the process of the systemic immunoglobulin production, although more in-depth studies are needed to explain these differences.

As we all know, *T. gondii* is one of the infectious agents causing early embryonic problems such as abortion, stillbirth, mummification, and death (Dubey, 2009). *T. gondii* has been considered a potential factor for reproductive failures in domestic animals worldwide (Nayeri et al., 2021). In this study, the domestic donkeys with a history of abortion have been found to demonstrate a significantly higher *T. gondii* seroprevalence compared to those without a history of abortion (**Table 2**). So, effective control measures and strategies are needed for reducing the rate of abortion in domestic donkeys as well as reducing the economic damage to the livestock industry.

Cats, as the final hosts of this parasite, excrete oocysts *via* their feces infecting the intermediate hosts such as the domestic animals (Dubey, 2004). The presence of cats in the animals’ habitat has been strongly associated with the prevalence of the anti-*T. gondii* antibodies (Moreira et al., 2019). In this study, the presence of a cat in the house was found to be a significant risk factor for *T. gondii* seropositivity among these tested domestic donkeys (*p* = 0.008; adjusted OR = 2.36; 95% CI: 1.26–4.44) (**Table 3**). Moreover, the tested domestic donkeys were collected from the rural areas, thus, the number of feral cats may be certainly large. Therefore, it is important to effectively bar cats out of the donkey’s habitat to reduce the incidence of infection.

Exploring the transmission route of toxoplasmosis infection in donkeys can provide important suggestions for preventing and treating toxoplasmosis. Undoubtedly, considering the dietary habits of herbivores, they are most likely to contract the infection by ingesting the oocysts that existed in their environment because feline is the final host of *T. gondii* discharging oocysts into the environment. Furthermore, some external forces such as wind, rain, and surface water can facilitate its diffusions in the environment. Although the source of water and source of fodder were not evaluated as the potential risk factors in the present study, these have been identified as the risk factors associated with *T. gondii* infection in domestic animals, such as cow, goat, sheep, and equids (Dubey et al., 2014; Gazzonis et al., 2019; Moreira et al., 2019). Thus, more future studies should be conducted for detecting the *T. gondii* oocysts in their environment for further assessment of the risk of infection.

In the present study, an available commercial validated ELISA kit was employed to test the serum–milk pairs and an optimal agreement was obtained between the results of the two biological matrices. In this case, it is easier and less expensive to collect the milk samples rather than collecting the serum samples. Moreover, collecting milk is less irritating to the animals. Thus, during the routine disease screening of toxoplasmosis at the individual, herd, and farm levels, this method should be considered for the first round of screening (Schares et al., 2004). However, more studies are needed for supporting the hypothesis of parasite transmission *via* the ingestion of raw milk or dairy products, including molecular diagnosis and biological methods.

Although this is the first study detecting the antibodies against *T. gondii* in donkey milk in China, two main limitations cannot be neglected. Firstly, the serum and milk samples were not respectively collected on a different phase of lactation from the same objects. Thus, the concordance between the serum and milk samples may be affected by some objective factors. Secondly, only serological tests were conducted in the present study. The diagnosis of toxoplasmosis merely based on serological tests is ineffective and insufficient. The serological results require a confirmatory diagnostic method that is based on directly demonstrating the parasite in the tissues or biological fluids by tissue culture or mouse inoculation. Thus, more studies should be conducted to verify the current results, including the isolation of the live organisms and more rigorous and standard sampling schemes.

Given the present results, health instruction from the health authorities must be implemented and distributed to the consumers of the animals’ milk. Boiling or pasteurization are recommended procedures for eliminating the risk of transmission of *T. gondii*. In addition, more studies should be carried out to evaluate the quantity and viability of *T. gondii* eliminated in the donkey’s milk. There is an immense need for some studies based on natural infections, especially in the rural or some individual farmers because they are habituated to consuming raw donkey milk. Both priority and special concerns should be focused on the most vulnerable consumer groups, including the immunocompromised patients, the aged, and babies with milk allergies. Moreover, heat treatment of the milk is strongly recommended before consumption.

## Data Availability Statement

The original contributions presented in the study are included in the article/supplementary material. Further inquiries can be directed to the corresponding authors.

## Ethics Statement

All procedures involving animals were approved by the Animal Care and Ethic Committee of Jilin Academy of Agricultural Sciences. Written informed consent was obtained from the owners for the participation of their animals in this study.

## Author Contributions

LC: Methodology, formal analysis, and writing—original draft. Z-JZ: Conceptualization, methodology, and writing—review and editing. Q-FM: Conceptualization and writing—review and editing. All authors contributed to the article and approved the submitted version.

## Funding

This research was supported by Basic Scientific Research Projects of Jilin Academy of Agricultural Sciences (KYJF2021ZR016); the Funding Program for High-Level Scientific and Technological Innovation Talents introduced by scientific research institutes of Jilin province (project no. 2018001); and the 68th General Grant of China Postdoctoral Science Foundation (project no. 2020M681063).

## Conflict of Interest

The authors declare that the research was conducted in the absence of any commercial or financial relationships that could be construed as a potential conflict of interest.

## Publisher’s Note

All claims expressed in this article are solely those of the authors and do not necessarily represent those of their affiliated organizations, or those of the publisher, the editors and the reviewers. Any product that may be evaluated in this article, or claim that may be made by its manufacturer, is not guaranteed or endorsed by the publisher.

## References

[B1] Azab M. E. Kamel A. M. Makled K. M. Khattab H. el-Zayyat E. A. Abo-Amer E. A.. (1992). Naturally Occurring Toxoplasma Antibodies in Serum and Milk of Lactating Women. J. Egypt. Soc. Parasitol. 22, 561–568. 1500798

[B2] Bezerra M. J. Kim P. C. Moraes É.P. Sá S. G. Albuquerque P. P. Silva J. G.. (2015). Detection of *Toxoplasma gondii* in the Milk of Naturally Infected Goats in the Northeast of Brazil. Transbound Emerg. Dis. 62, 421–424. doi: 10.1111/tbed.12160 24034351

[B3] Boughattas S. (2017). *Toxoplasma* Infection and Milk Consumption: Meta-Analysis of Assumptions and Evidences. Crit. Rev. Food. Sci. Nutr. 57, 2924–2933. doi: 10.1080/10408398.2015.1084993 26467987

[B4] Camossi L. G. Greca-Júnior H. Corrêa A. P. Richini-Pereira V. B. Silva R. C. Da Silva A. V.. (2011). Detection of *Toxoplasma gondii* DNA in the Milk of Naturally Infected Ewes. Vet. Parasitol. 177, 256–261. doi: 10.1016/j.vetpar.2010.12.007 21216534

[B5] Cong W. Chen L. Shan X. F. Qian A. D. Meng Q. F. (2018). First Genetic Characterization of *Toxoplasma gondii* Infection in Donkey Meat Slaughtered for Human Consumption in Shandong Province, Eastern China. Infect. Genet. Evol. 61, 1–3. doi: 10.1016/j.meegid.2018.03.008 29530661

[B6] Dehkordi F. S. Borujeni M. R. Rahimi E. Abdizadeh R. (2013). Detection of *Toxoplasma gondii* in Raw Caprine, Ovine, Buffalo, Bovine, and Camel Milk Using Cell Cultivation, Cat Bioassay, Capture ELISA, and PCR Methods in Iran. Foodborne. Pathog. Dis. 10, 120–125. doi: 10.1089/fpd.2012.1311 23441913

[B7] Dubey J. P. (2004). Toxoplasmosis - A Waterborne Zoonosis. Vet. Parasitol. 126, 57–72. doi: 10.1016/j.vetpar.2004.09.005 15567579

[B8] Dubey J. P. (2009). History of the Discovery of the Life Cycle of *Toxoplasma gondii* . Int. J. Parasitol. 39, 877–82. doi: 10.1016/j.ijpara.2009.01.005 19630138

[B9] Dubey J. P. (2010). Toxoplasmosis of Animals and Humans. Boca Raton, FL: CRC Press.

[B10] Dubey J. P. Ness S. L. Kwok O. C. Choudhary S. Mittel L. D. Divers T. J. (2014). Seropositivity of *Toxoplasma gondii* in Domestic Donkeys (*Equus Asinus*) and Isolation of *T. gondii* From Farm Cats. Vet. Parasitol. 199, 18–23. doi: 10.1016/j.vetpar.2013.09.027 24140163

[B11] Gazzonis A. L. Zanzani S. A. Stradiotto K. Olivieri E. Villa L. Manfredi M. T. (2018). *Toxoplasma gondii* Antibodies in Bulk Tank Milk Samples of Caprine Dairy Herds. J. Parasitol. 104, 560–565. doi: 10.1645/17-44 29906217

[B12] Gazzonis A. L. Zanzani S. A. Villa L. Manfredi M. T. (2019). *Toxoplasma gondii* in Naturally Infected Goats: Monitoring of Specific IgG Levels in Serum and Milk During Lactation and Parasitic DNA Detection in Milk. Prev. Vet. Med. 170, 104738. doi: 10.1016/j.prevetmed.2019.104738 31421505

[B13] Haridy F. M. Saleh N. M. Khalil H. H. Morsy T. A. (2010). Anti-*Toxoplasma gondii* Antibodies in Working Donkeys and Donkey's Milk in Greater Cairo, Egypt. J. Egypt. Soc. Parasitol. 40, 459–464. 21246953

[B14] Iacobucci E. Taus N. S. Ueti M. W. Sukhbaatar L. Bastsukh Z. Papageorgiou S.. (2019). Detection and Genotypic Characterization of *Toxoplasma gondii* DNA Within the Milk of Mongolian Livestock. Parasitol. Res. 118, 2005–2008. doi: 10.1007/s00436-019-06306-w 30982139 PMC6521982

[B15] Koethe M. Schade C. Fehlhaber K. Ludewig M. (2017). Survival of *Toxoplasma gondii* Tachyzoites in Simulated Gastric Fluid and Cow's Milk. Vet. Parasitol. 233, 111–114. doi: 10.1016/j.vetpar.2016.12.010 28043380

[B16] Li Y. Fan Y. Shaikh A. S. Wang Z. Wang D. Tan H. (2020). Dezhou Donkey (*Equus Asinus*) Milk a Potential Treatment Strategy for Type 2 Diabetes. J. Ethnopharmacol. 246, 112221. doi: 10.1016/j.jep.2019.112221 31494203

[B17] Li Q. Li M. Zhang J. Shi X. Yang M. Zheng Y.. (2020). Donkey Milk Inhibits Triple-Negative Breast Tumor Progression and Is Associated With Increased Cleaved-Caspase-3 Expression. Food. Funct. 11, 3053–3065. doi: 10.1039/C9FO02934F 32191229

[B18] Luoyizha W. Wu X. Zhang M. Guo X. Li H. Liao X. (2020). Compared Analysis of Microbial Diversity in Donkey Milk From Xinjiang and Shandong of China Through High-Throughput Sequencing. Food. Res. Int. 2137, 109684. doi: 10.1016/j.foodres.2020.109684 33233260

[B19] Mancianti F. Nardoni S. Papini R. Mugnaini L. Martini M. Altomonte I.. (2014). Detection and Genotyping of *Toxoplasma gondii* DNA in the Blood and Milk of Naturally Infected Donkeys (*Equus Asinus*). Parasitol. Vectors. 7, 165. doi: 10.1186/1756-3305-7-165 PMC3985582 24708691

[B20] Martini M. Altomonte I. Mancianti F. Nardoni S. Mugnaini L. Salari F. (2014). A Preliminary Study on the Quality and Safety of Milk in Donkeys Positive for *Toxoplasma gondii* . Animal. 8, 1996–1998. doi: 10.1017/S1751731114001980 25118707

[B21] Meng Q. F. Li D. Yao G. Z. Zou Y. Cong W. Shan X. F. (2018). Seroprevalence of *Toxoplasma gondii* Infection and Variables Associated With Seropositivity in Donkeys in Eastern China. Parasite. 25, 66. doi: 10.1051/parasite/2018066 30526821 PMC6289070

[B22] Miao Q. Wang X. She L. N. Fan Y. T. Yuan F. Z. Yang J. F.. (2013). Seroprevalence of *Toxoplasma gondii* in Horses and Donkeys in Yunnan Province, Southwestern China. Parasitol. Vectors. 6, 168. doi: 10.1186/1756-3305-6-168 PMC3679964 23742078

[B23] Montoya J. G. Liesenfeld O. (2004). Toxoplasmosis. Lancet. 363, 1965–1976. doi: 10.1016/S0140-6736(04)16412-X 15194258

[B24] Moreira T. R. Sarturi C. Stelmachtchuk F. N. Andersson E. Norlander E. de Oliveira F. L. C.. (2019). Prevalence of Antibodies Against *Toxoplasma gondii* and Neospora Spp. In Equids of Western Para, Brazil. Acta Trop. 189, 39–45. doi: 10.1016/j.actatropica.2018.09.023 30267659

[B25] Nayeri T. Sarvi S. Moosazadeh M. Daryani A. (2021). Global Prevalence of *Toxoplasma gondii* Infection in the Aborted Fetuses and Ruminants That had an Abortion: A Systematic Review and Meta-Analysis. Vet. Parasitol. 290, 109370. doi: 10.1016/j.vetpar.2021.109370 33550003

[B26] Perrucci S. Guardone L. Altomonte I. Salari F. Nardoni S. Martini M.. (2021). Apicomplexan Protozoa Responsible for Reproductive Disorders: Occurrence of DNA in Blood and Milk of Donkeys (*Equus Asinus*) and Minireview of the Related Literature. Pathogens. 10, 111. doi: 10.3390/pathogens10020111 33499205 PMC7912328

[B27] Petruzzelli A. Amagliani G. Micci E. Foglini M. Di Renzo E. Brandi G.. (2011). Prevalence Assessment of Coxiella burnetii and Verocytotoxin-Producing Escherichia coli in Bovine Raw Milk Through Molecular Identification. Food. Control. 32, 532–536. doi: 10.1016/j.foodcont.2013.01.041

[B28] Pinto-Ferreira F. Caldart E. T. Pasquali A. K. S. Mitsuka-Breganó R. Freire R. L. Navarro I. T. (2019). Patterns of Transmission and Sources of Infection in Outbreaks of Human Toxoplasmosis. Emerg. Infect. Dis. 25, 2177–2182. doi: 10.3201/eid2512.181565 31742524 PMC6874273

[B29] Powell C. C. Brewer M. Lappin M. R. (2001). Detection of *Toxoplasma gondii* in the Milk of Experimentally Infected Lactating Cats. Vet. Parasitol. 102, 29–33. doi: 10.1016/S0304-4017(01)00521-0 11705649

[B30] Radon K. Windstetter D. Eckart J. Dressel H. Leitritz L. Reichert J.. (2004). Farming Exposure in Childhood, Exposure to Markers of Infections and the Development of Atopy in Rural Subjects. Clin. Exp. Allergy 34, 1178–1183. doi: 10.1111/j.1365-2222.2004.02005.x 15298556

[B31] Rehman F. Shah M. Ali A. Ahmad I. Sarwar M. T. Rapisarda A. M. C.. (2020). Unpasteurised Milk Consumption as a Potential Risk Factor for Toxoplasmosis in Females With Recurrent Pregnancy Loss. J. Obstet. Gynaecol. 40, 1106–1110. doi: 10.1080/01443615.2019.1702630 32013639

[B32] Saad N. M. Hussein A. A. A. Ewida R. M. (2018). Occurrence of *Toxoplasma gondii* in Raw Goat, Sheep, and Camel Milk in Upper Egypt. Vet. World. 11, 1262–1265. doi: 10.14202/vetworld.2018.1262-1265 30410231 PMC6200564

[B33] Schares G. Bärwald A. Staubach C. Wurm R. Rauser M. Conraths F. J.. (2004). Adaptation of a Commercial ELISA for the Detection of Antibodies Against *Neospora Caninum* in Bovine Milk. Vet. Parasitol. 120, 55–63. doi: 10.1016/j.vetpar.2003.11.016 15019143

[B34] Tafaro A. Magrone T. Jirillo F. Martemucci G. D'Alessandro A. G. Amati L.. (2007). Immunological Properties of Donkey's Milk: Its Potential Use in the Prevention of Atherosclerosis. Curr. Pharm. Des. 13, 3711–3717. doi: 10.2174/138161207783018590 18220810

[B35] Tenter A. M. M. Heckeroth A. R. R. Weiss L. M. M. (2000). *Toxoplasma gondii*: From Animals to Humans. Int. J. Parasitol. 30, 1217–1258. doi: 10.1016/S0020-7519(00)00124-7 11113252 PMC3109627

[B36] Yang N. Mu M. Y. Yuan G. M. Zhang G. X. Li H. K. He J. B. (2013). Seroprevalence of *Toxoplasma gondii* in Slaughtered Horses and Donkeys in Liaoning Province, Northeastern China. Parasitol. Vectors. 6, 140. doi: 10.1186/1756-3305-6-140 PMC3659062 23680297

[B37] Zhang X. Jiang B. Ji C. Li H. Yang L. Jiang G.. (2021). Quantitative Label-Free Proteomic Analysis of Milk Fat Globule Membrane in Donkey and Human Milk. Front. Nutr. 8, 670099. doi: 10.3389/fnut.2021.670099 34239890 PMC8258387

[B38] Zhang X. X. Shi W. Zhang N. Z. Shi K. Li J. M. Xu P.. (2017). Prevalence and Genetic Characterization of *Toxoplasma gondii* in Donkeys in Northeastern China. Infect. Genet. Evol. 54, 455–457. doi: 10.1016/j.meegid.2017.08.008 28807857

